# Therapeutic Vaccines for Chronic Viral Infections: From Immune Modulation to Clinical Translation

**DOI:** 10.3390/vaccines14060507

**Published:** 2026-06-04

**Authors:** Zhuang Li, Yuan Zhang, Yiyang Zheng, Hongyu Wang, Chenyang Xu, Qing He

**Affiliations:** 1 State Key Laboratory of Drug Regulatory Sciences, National Institutes for Food and Drug Control, Beijing 102629, China; lizhuang0422@163.com (Z.L.);; 2 School of Biopharmacy, China Pharmaceutical University, Nanjing 211198, China

**Keywords:** therapeutic vaccines, chronic viral infections, functional cure, T-cell exhaustion, combination therapy, hepatitis B virus, hepatitis C virus, human immunodeficiency virus, human papillomavirus, Epstein–Barr virus

## Abstract

Therapeutic vaccines are a key strategy to achieve the goal of “functional cure” of chronic viral infections, including hepatitis B virus (HBV), hepatitis C virus (HCV), human immunodeficiency virus (HIV), human papillomavirus (HPV), and Epstein–Barr virus (EBV). Various platforms (such as viral vectors, nucleic acid vaccines, recombinant proteins, etc.) have successfully induced strong virus-specific T-cell responses in early trials, but their clinical efficacy is still limited by the immunosuppressive environment formed by the host. The core bottlenecks are severe T-cell exhaustion, viral immune escape, and various forms of local immune tolerance. Therefore, the field is moving toward combination therapies, including reduction of viral load, targeting of immune activation, and inhibition of inhibitory signaling pathways. This article summarizes the preclinical and clinical progress of therapeutic vaccines in the past decade, analyzes the major challenges in vaccine development, and discusses the future development directions in this field.

## 1. Introduction

Chronic viral infections have imposed a significant burden on global public health. The main pathogens causing these infections include hepatitis B virus (HBV), hepatitis C virus (HCV), human immunodeficiency virus (HIV), high-risk human papillomavirus (HPV), and Epstein–Barr virus (EBV) [1,2,3,4,5,6]. The persistent infection of these viruses leads to pathogen-specific pathological progression, which eventually triggers a series of life-threatening terminal diseases, such as liver cirrhosis, acquired immune deficiency syndrome (AIDS), and virus-related malignant tumors (such as hepatocellular carcinoma, cervical cancer, and nasopharyngeal carcinoma). From an immunologic perspective, chronic viral infections are characterized by persistent low-level specific immune responses of the host against pathogens [6,7,8,9]. This fundamentally reflects the immune evasion characteristics of these viruses, that is, their evolved ability to evade the attack of the body’s immune system, so that they can evade or actively suppress the host’s immune surveillance and thus survive in the host for a long time [10].

Current antiviral intervention measures have limitations in treating chronic viral infections [11]. For HBV and HIV infections, existing antiviral drugs can effectively inhibit viral replication, but they cannot eliminate the viral reservoir that exists in cells for a long time [12,13]. Regarding HCV infection, although direct-administered antiviral drugs (DAAs) can achieve a relatively high viral clearance rate, they cannot induce persistent protective immune memory to prevent reinfection. For viruses such as human papillomavirus (HPV) and Epstein–Barr virus (EBV), there is still a lack of effective antiviral agents that can specifically clear latent infection [14]. Therefore, the existing drug interventions carried out through the host immune system are usually insufficient to achieve sustained and stable control effects after the virus control stops, and thus cannot achieve functional cure.

To address these unmet clinical needs, therapeutic vaccines aim to restore the host-specific antiviral immune response, thus becoming a key research strategy for achieving functional cure [15]. Preventive vaccines mainly establish a protective immune barrier (for example, by inducing neutralizing antibodies) to prevent initial infections, while therapeutic vaccines focus on activating or reshaping the cellular immune response in cases of infection [16]. Currently, research in this field is moving towards more systematic immune intervention models, including verifying and applying efficient antigen delivery platforms, optimizing antigen design and immune response characteristics, and strategies that combine vaccines with immunomodulatory methods. These advancements aim to more effectively reactivate the impaired immune system and provide new possibilities for the clinical management of various chronic viral infections.

In this review, we discuss recent advances in preclinical research and clinical translation of therapeutic vaccines targeting major chronic viral infections, explore the key scientific challenges currently facing this field, and provide perspectives on future directions for achieving clinical functional cure.

## 2. The Mechanism of Therapeutic Vaccines Inducing Antiviral Immunity

**Figure 1:**
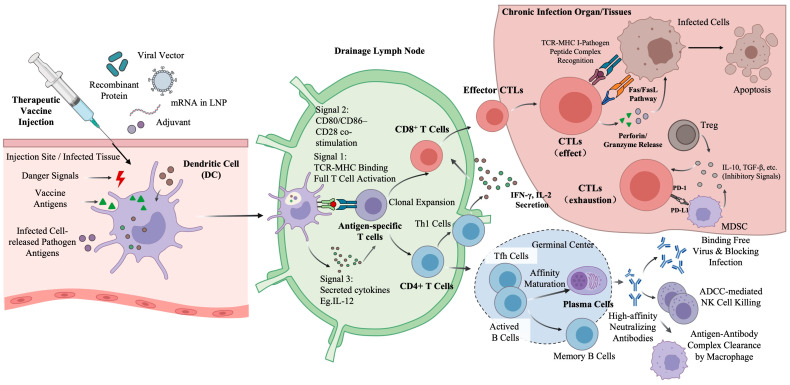
The antiviral immune mechanism of therapeutic vaccines.

The core mechanism of therapeutic vaccines is to reverse T cell exhaustion and central/peripheral immune tolerance caused by chronic infection. As shown in Figure 1, it reconstructs the host antiviral immune response by reshaping the antigen presentation and T-cell activation pathways.

First, vaccine delivery systems (e.g., viral vectors, lipid nanoparticles, or recombinant proteins) target antigen delivery to antigen-presenting cells (APCs). Local innate dendritic cells (DCs) take up antigens and mature in response to adjuvant or vector-derived pathogen-associated molecular patterns (PAMPs), which up-regulate the expression of MHC molecules and costimulatory molecules. Mature DCs migrate to the drainage lymph nodes and simultaneously activate CD4^+^ T cells through the MHC class II pathway and load exogenous antigens to MHC class I molecules through the cross-presentation pathway, thereby overcoming the activation barrier of CD8^+^ T cells in chronic infection.

Full activation of T cells relies on a “three-signal model”. Signal 1 is provided by TCR recognition of the pMHC complex; Signal 2 is provided by CD80/CD86 binding to CD28; Signal 3, which is provided by cytokines such as IL-12 secreted by DC, determines the fate of T cell differentiation. Activated CD4^+^ T cells differentiated into Th1 subsets and secreted IFN-γ and IL-2. The former enhanced CTL killing function, and the latter promoted T cell clonal expansion. Some CD4^+^ T cells differentiate into T follicular helper (Tfh) cells, which drive the germinal center reaction and promote the affinity maturation of B cells to form memory B cells and long-acting plasma cells.

In addition, therapeutic vaccines induce functional immune memory. Endogenous antigens and damage-associated molecular patterns (DAMPs) released during the effector phase can be re-taken up by DCS, forming a cascade of amplification effects. This process promotes the formation of central memory T cells (Tcm) and tissue-resident memory T cells (TRM), realizes long-term immune surveillance of pathogens, and lays the foundation for functional cure of chronic infection [17].

Although Figure 1 shows the general process of vaccine-induced antiviral immunity, this process should be understood in the context of chronic infection rather than as a primary immune response in an immunologically naïve host. In patients with chronic viral infection, virus-specific immune responses have usually been primed for a long time, but these responses are weakened or reshaped by persistent antigen exposure, viral reservoirs, and tissue-specific immune tolerance. Therefore, the key issue for therapeutic vaccines is not simply to induce antigen-specific T cells, but to restore effective antiviral immunity in an immune system that has already been impaired by long-term viral persistence.

Therapeutic vaccines are designed to restore virus-specific immunity in hosts with established chronic infection. Vaccine antigens are presented by dendritic cells to activate CD4^+^ and CD8^+^ T cells. CD8^+^ T cells differentiate into effector cytotoxic T lymphocytes that migrate to infected tissues and mediate infected-cell killing, whereas CD4^+^ T cells support Th1 responses and T follicular helper cell-mediated B cell activation, leading to antibody production and immune memory. In chronic infection, these processes are constrained by persistent viral reservoirs, long-term antigen exposure, tissue-specific immune tolerance, and T cell exhaustion.

## 3. Current Progress in Preclinical and Clinical Research

Preclinical and clinical translational research on therapeutic vaccines for chronic viral infections has been accelerating in recent years, and its technological evolution follows a highly convergent iterative path. In the dimension of antigen design, the focus of research has gradually shifted from the early single antigen strategy to the rational antigen design and optimization under the guidance of multi-antigen combination, precise targeting of conserved epitopes and structural vaccinology. The core goal of this prohibition is to efficiently induce CTL responses with breadth and versatility to overcome the immune escape caused by viral mutation. At the delivery system level, new platform technologies such as viral vectors, nucleic acid vaccines (DNA/mRNA), synthetic peptides and virus-like particles (VLP) are becoming more mature. With their high lymphatic homing ability and antigen presentation efficiency, they have shown great potential to reconstitute immune homeostasis and break peripheral immune tolerance caused by chronic infection. In terms of clinical transformation strategy, given that it is difficult for a single vaccine intervention to completely reverse the deep immunosuppressive microenvironment established by long-term infection, the current strategy has shifted from monotherapy to combination therapy, aiming to reshape the antiviral immunity through synergistic effects. The following text will respectively elaborate on the research progress of therapeutic vaccines against different viruses.

### 3.1. HBV

HBV is a hepatotropic DNA virus defined by a partially double-stranded circular genome. It drives the development of chronic infection via the long-term persistence of covalently closed circular DNA (cccDNA) within the nuclei of hepatocytes [18,19]. Existing nucleos(t)ide analogues can only inhibit viral replication, but cannot eradicate cccDNA or reverse this immunodepletion, making it difficult to achieve a functional cure [20]. Therefore, therapeutic vaccines aim to restore the host’s specific anti-HBV immune response, and they are an important means to achieve effective control of chronic hepatitis B (CHB). In order to effectively break the immune tolerance state of CHB patients, the design of therapeutic vaccines has shifted from the traditional single antigen strategy (such as targeting only the hepatitis B surface antigen) to a multi-antigen combination strategy. By incorporating core antigen (HBcAg), polymerase (Pol), surface antigen (HBsAg), and other viral components, these vaccines aim to elicit strong cellular immunity and maximize the breadth and polyfunctionality of CTL responses [21].

After the multi-antigen targeting strategy was established, the focus of preclinical research turned to the iteration of delivery systems and adjuvant technology to specifically break liver immune tolerance and enhance antigen cross-presentation. The core challenge of immune intervention against viral infections such as chronic hepatitis B is to reverse the depletion state of antigen-specific T cells and reshape the intrahepatic immune microenvironment. In the optimization of adjuvant systems, research has focused on reshaping the immune response through pattern recognition receptor agonists. For example, CLB-3000, a recombinant protein vaccine combining modified HBsAg with a saponin-based adjuvant (TQL-1055), successfully reduced circulating HBsAg and cleared infected cells in HBV mouse models [22]. On this basis, the combined adjuvant strategy based on Toll-like receptor (TLR) agonists shows better potential. Recent studies confirm that combining HBsAg with a CpG oligodeoxynucleotide-containing dual adjuvant (e.g., CpG 1018S/QS-21) significantly downregulates T-cell exhaustion markers (TIM-3 and TIGIT) and achieves profound viral suppression in AAV-HBV mouse models [23]. In the construction of delivery platforms, different technical routes are focused on efficient delivery of antigens into the MHC-I pathway to directly activate CD8^+^ T cells. The viral vector platform (such as MVA and chimpanzee adenovirus ChAdOx1) takes advantage of its in situ expression property within host cells, effectively circumventing the efficiency bottleneck of extracellular antigen presentation. For instance, the MVA vector-based TherVacB vaccine induced robust cytotoxic T-cell responses in mouse models, effectively controlling intrahepatic HBV DNA replication [24]. Similarly, the vaccine based on the chimpanzee adenovirus vector (ChAdOx1-HBV) demonstrated excellent immunogenicity. Pre-clinical studies indicated that ChAdOx1-HBV could break through the immune tolerance state of mice and induce a higher proportion of antigen-specific T cells [25]. Nucleic acid vaccine platforms also show good efficacy in preclinical evaluations. A recent study shows that mRNA-LNP vaccination can eliminate serum HBsAg and significantly reduce intrahepatic cccDNA levels in HBV transgenic mice [26]. Furthermore, DNA vaccines combined with in vivo electroporation technology have effectively overcome the bottleneck of low traditional plasmid delivery efficiency. Recent studies confirm that DNA vaccines encoding hepatitis B virus core protein and polymerase protein by electroporation can safely elicit strong cellular immune responses based on Th1 cell subsets in experimental animal models [27]. Although these candidate vaccines can achieve effective viral suppression in preclinical models, existing animal models have certain limitations. They do not fully reproduce what results from long-term chronic human infection, severe immunodepleting and a complex hepatic inhibitory microenvironment [28]. Therefore, the actual immunogenicity and clinical antiviral efficacy of these candidate vaccines require rigorous validation in clinical trials involving patients with chronic hepatitis B.

Benefiting from the positive data of preclinical studies, a variety of therapeutic hepatitis B vaccines using different technical platforms have successively entered clinical evaluation, including recombinant proteins, virus-like particles (VLPs), DNA plasmids, peptides, and viral vectors. Taken together, the HBV clinical studies listed in Table 1 suggest that therapeutic vaccines can induce HBV-specific humoral and cellular immune responses, but vaccination alone rarely leads to durable functional cure. Stronger clinical signals are generally observed when vaccines are combined with antigen-reduction strategies, PEG-IFNα, or immune checkpoint modulation, especially in patients with lower baseline HBsAg levels. This indicates that persistent HBsAg burden, intrahepatic immune tolerance, and exhaustion of HBV-specific T cells remain major barriers to efficacy in CHB patients. Recurrent design features in HBV studies include multi-antigen vaccine constructs, heterologous prime–boost regimens, and combination strategies designed to reduce antigen load before or during immune reactivation.

Based on these overall patterns, current clinical trials have shown that candidate vaccines based on VLPs and recombinant protein platforms are relatively advanced in research and development due to their good safety and immunogenicity. In contrast, nucleic acid and viral vector platforms have shown great potential for eliciting potent and specific T cell responses. In VLP-based platforms, candidates such as NASVAC/ABX203 (expressing HBcAg and HBsAg) have advanced to Phase 3 clinical trials. Its unique intranasal and subcutaneous administration route is specifically designed to elicit both mucosal and systemic immunity. Long-term follow-up data from the NASVAC phase III clinical trial demonstrated sustained suppression of hepatitis B virus (HBV) DNA at 2, 3, and 5 years post-treatment. In the majority of patients, the viral load was undetectable or remained below baseline levels, with liver function markers (ALT) normal or near-normal, alongside significant HBeAg seroconversion [29]. Concurrently, the study of the recombinant protein vaccine BRII-179 (VBI-2601) confirmed the successful induction of humoral immunity against S, Pre-S1, and Pre-S2 antigens, alongside the critical restoration of IFN-γ-producing specific T cell responses [30]. In parallel, early clinical trials are actively evaluating the ability of nucleic acid and protein platforms to stimulate cellular immunity. For example, DNA vaccines such as JNJ-64300535 and INO-1800, which encode highly conserved regions such as core proteins and polymerase proteins, have shown safety and immunogenicity in patients with viral suppression and continuous use of standard nucleoside analogues (such as TDF, ETV) [31,32]. Additionally, the viral vector platform showed better T cell priming ability, especially to stimulate stronger CD8^+^ T cell immunity after heterologous sequential immunization [33]. Adenovirus-based constructs, such as T101 (TG1050) and VRON-0200, are designed to deliver multiple hepatitis B virus antigens to maximize the breadth of T cells [34,35]. A typical example is VTP-300 (using a chimpanzee adenovirus vector ChAdOx1-HBV for the initial immunization and an MVA-HBV vector for the booster immunization), which successfully induced sustained antigen-specific CD4^+^ and CD8^+^ T cell responses [36]. In patients treated with VTP-300, especially those with low baseline HBsAg levels (<50 IU/mL), we observed a sustained and significant reduction in HBsAg (more than 0.7 log units).

Although single therapeutic vaccines have shown potential in inducing specific immune responses, existing clinical evidence consistently shows that vaccine intervention alone is not enough to completely reverse the highly immunosuppressive liver microenvironment that has developed in patients with CHB. Therefore, the development of reasonable combination treatment strategies has become a key direction for clinical translation. On the one hand, by combining with pegylated interferon-α (PEG-IFNα) to synergistically activate innate and adaptive immunity, clinical data show that patients who respond to the BRII-179 vaccine have a significant increase in HBsAg clearance when they are subsequently treated with PEG-IFNα [37]. On the other hand, the combination of small interfering RNA (siRNA) or neutralizing monoclonal antibodies for Antigen Reduction has become an important prerequisite to reshape the immune microenvironment for immune paralysis caused by persistent high antigen load [38]. Multiple ongoing clinical trials, such as JNJ-64300535 in combination with siRNA JNJ-73763989 and VRON-0200 in combination with VIR-2218 and VIR-3434, are working to validate this strategy [31,35]. Notably, Phase II clinical data revealed that patients who previously responded to BRII-179 achieved an impressive HBsAg clearance rate of 61% when subsequently treated with a combination of PEG-IFNα and the siRNA BRII-835 for up to 48 weeks. Among those maintaining high anti-HBs titers, the clearance rate reached 91% [39]. In addition, in response to the phenomenon of “re-depletion” of vaccine-derived T cells after entering the PD-L1-rich liver microenvironment, current clinical protocols have begun to integrate immune checkpoint inhibitors to block inhibitory signals. For example, the clinical trial of VTP-300 innovated the incorporation of a low-dose PD-1 inhibitor (nivolumab), which not only effectively prolonged the survival time of cytotoxic T cells, but also prevented the re-loss of function while maintaining their targeted killing ability against cccDNA-positive hepatocytes [36].

**Table 1:** Clinical study of therapeutic vaccines for HBV infection (past decade).

Name	Vaccine Type	HBV Antigens Within Vaccine	Combination Therapy	NCT Number	Phase	Recruitment Status	Ref.
NASVAC/ABX203	VLPs	HBcAg/HBsAg	/	NCT01374308	3	Unknown status	[40]
BRII-179 (VBI-2601)	recombinant protein	Pre-S1/Pre-S2/HBsAg	IFN-α BRII-835	NCT04749368	2	Completed	[41]
			PEG-IFN-α BRII-835	NCT06491563	2	Activate Not, Recruiting	[42]
			PEG-IFN-α BRII-835	NCT06650852	2	Recruiting	[43]
ePA-44	polypeptide	Epitopes of HBsAg, Pre-S2, HBcAg, Pol	entecavir	NCT01326546	2	Completed	[44]
CVI-HBV-002	recombinant protein	HBsAg	TDF	NCT04289987	2	Completed	[45]
VTP-300	AdV/MVA	HBcAg/Pol/HBsAg (Pre-S1/Pre-S2/S)	nivolumab	NCT05343481	2	Activate Not, Recruiting	[46]
ISA104	polypeptide	Synthetic Long Peptides of HBcAg, Po	/	NCT05841095	2	Unknown status	[47]
GS-4774	Inactivated yeast	HBcAg/HBsAg/HBx	TDF	NCT02174276	2	Completed	[48]
HepTcell	polypeptide	Conserved epitopes of HBcAg, Pol, HBsAg	/	NCT04684914	2	Terminated	[49]
GSK3528869A	AdV/MVA	HBcAg/HBsAg	/	NCT03866187	1/2	Terminated	[50]
TherVacB	recombinant protein MVA	Pol/HBcAg/HBsAg	/	NCT06513286	1/2	Enrolling by invitation	[51]
T101 (TG1050)	AdV	Pol/HBcAg/HBsAg domains	/	NCT04168333	1	Completed	[34]
			/	NCT02428400	1	Completed	[52]
JNJ-64300535	DNA	HBcAg/Pol	Nas	NCT03463369	1	Completed	[53]
			JNJ-73763989/ETV/TDF/TAF	NCT05123599	1	Completed	[31]
VRON-0200-AdC6/AdC7	AdV	HBcAg/Pol (Fused to Glycoprotein D)	VIR-3434/VIR-2218	NCT06070051	1	Completed	[35]
INO-1800	DNA	HBsAg/HBcAg	INO-1800/Nas	NCT02431312	1	Completed	[32]
HB-110	DNA	HBeAg	ETV	NCT01641536	1	Completed	[54]
ChAdOx1-HBV	Adenovirus	Pol/HBcAg/HBsAg	/	NCT04297917	1	Completed	[55]

### 3.2. HCV

Hepatitis C virus (HCV), as a positive-stranded single-stranded RNA virus with high genetic diversity, is mainly transmitted through blood [56]. Although direct-acting antivirals (DAAs), which target viral nonstructural proteins, have been able to achieve 90% to 95% sustained virologic response (SVR) in the vast majority of infected patients with genotypes [57], this therapy still has significant clinical limitations. Firstly, even if these medications eliminate the virus, they cannot eliminate the danger of the development of liver cancer in the treated patient [58]. Secondly, due to the lack of proofreading function of viral RNA polymerase, the virus produces a high frequency of gene mutations and forms a “quasispecies” library during replication [59]. This high degree of genetic instability not only promotes the emergence of drug-resistant variants (such as the C316 mutation in the NS3 protease region and the S282T mutation in the NS5B region) that lead to treatment failure but also increases the risk of long-term recurrence [60]. In view of this, therapeutic vaccines that can induce long-lasting and broad-spectrum protective immune responses are regarded as important supplementary or alternative strategies to make up for the deficiencies of existing DAA therapies and ultimately achieve functional cure and even prevention of hepatitis C.

To date, the development of therapeutic vaccines proceeds in parallel with research on prophylactic vaccines—both efforts are fundamentally grounded in elucidating viral infection mechanisms and serve as critical testbeds for evaluating diverse vaccine platforms. In preclinical research, researchers have explored various HCV vaccine candidates based on viral vectors, recombinant inactivated viruses, virus-like particles (VLPs), nanoparticles, recombinant proteins, peptides, and DNA. E1 and E2 are the major targets of neutralizing antibodies (nAbs), and thus also the main targets for the development of vaccines [61,62]. A representative example is the candidate hepatitis C virus (HCV) vaccine based on the mrNA-lipid nanoparticle (mRNA-LNP) platform, which encodes a soluble HCV envelope glycoprotein E1 (sE1) and a modified E2 variant (sE2F442NYT). This variant was modified to reduce CD81 binding affinity. The vaccine induced enhanced cross-genotypic neutralizing antibodies and a strong Th1-biased cellular immune response, accompanied by substantial increases in interferon-γ (IFN-γ) and granzyme B levels [63]. Since HCV is easily mutated, vaccines targeting structural proteins often face the risk of immune escape, so the focus of research and development has gradually turned to highly conserved non-structural proteins (such as p7, NS2, NS3/NS4A, NS4B, NS5A and NS5B) [64]. These proteins are functionally critical and sequence-conserved during the viral replication cycle, and are ideal targets for T-cell-centered therapeutic vaccines designed to achieve immune clearance of infected hepatocytes by activating a broad-spectrum CD8^+^/CD4^+^ T cell response targeting conserved epitopes [65].

The clinical translation of HCV therapeutic vaccines remains at an early exploratory stage. Current studies mainly provide evidence of immunogenicity rather than clear clinical benefit. This situation is partly related to the current therapeutic landscape of HCV infection: because direct-acting antivirals (DAAs) already achieve high sustained virological response rates, the future role of HCV therapeutic vaccines may be more focused on inducing durable immune protection, preventing reinfection, or benefiting selected high-risk populations. The main limitations are the high genetic variability of HCV, immune escape, and the difficulty of defining vaccine-specific clinical endpoints in the DAA era. Accordingly, the current clinical pipeline is mainly composed of DNA plasmid and viral vector-based vaccine candidates, most of which are in phase I clinical trials (Table 2). Current trial designs therefore tend to focus on conserved nonstructural antigens and on platforms capable of inducing broad T cell responses.

Among these T-cell-centered immunization strategies, INO-8000-a, a DNA vaccine encoding HCV nonstructural proteins (NS3, NS4A, NS4B, and NS5A), has been evaluated in phase I trials; When combined with INO-9012, a plasmid encoding IL-12, it successfully induced HCV-specific CD8^+^ and CD4^+^ T cell responses in 89% and 82% of subjects, respectively, demonstrating the potential of this platform to stimulate cellular immunity [66]. In addition, heterologous prime vaccine-boost strategies using chimpanzee adenovirus type 3 (ChAd3) and modified vaccinia Ankara (MVA), engineered to express the same nonstructural antigen, also elicited high-frequency, multifunctional HCV-specific T cell responses in subjects [67]. These early clinical data not only validate the immunogenicity of targeting conserved nonstructural proteins but also provide a feasible clinical pathway for the development of more effective therapeutic vaccines against HCV.

Although the rapid mutation rate of HCV and the limits of the animal models for vaccine development are challenges, therapeutic vaccines are still of great clinical importance. They are of particular importance to decrease the chance of drug resistance and improve the current treatment [68,69,70]. In the context of the proofreading mechanisms of RNA polymerase, the development of vaccines requires the use of several antigenic components, of new delivery platforms such as virus-like particles, and of better adjuvant formulations. The idea is to act at the same time in order to induce a good production of neutralizing antibody and to activate also polyfunctional CD8^+^ T cell immunity [71,72]. Furthermore, by combining these vaccines with immunomodulators (including the use of PD-1 pathway blockers to counteract T cell dysfunction) or direct antiviral compounds, potential synergistic effects may be achieved, which could help eliminate infected liver cells and improve the regulation of the immune response [73]. Regarding the experimental model, although using chimpanzees is no longer feasible, new technologies are constantly emerging, such as the humanized mouse system through genetic modification and tissue culture techniques. These technologies are gradually providing a more clinically relevant platform for the preclinical evaluation of vaccines [74]. Notably, combination strategies of therapeutic vaccines with direct antivirals have been proposed and are currently in the validation stage for specific clinical situations, such as reducing the risk of drug-resistant strains when people who inject drugs become re-infected, or enhancing DAA therapy to reduce the incidence of hepatocellular carcinoma in patients with cirrhosis [71].

**Table 2:** Clinical study of therapeutic vaccines for HCV infection (past decade).

Name	Vaccine Type	HCV Antigens Within Vaccine	Combination Therapy	NCT Number	Phase	Recruitment Status	Ref.
Autologous DC-vaccines	DC cells	HCV-antigens (Core and NS3)	/	NCT03119025	1/2	Completed	[75]
HCVax™	lentiviral vector	several HCV antigens	/	NCT04318379	1	Unknown status	[76]
6INO-8000	DNA	HCV-antigens	INO-9012	NCT02772003	1	Activate Not, Recruiting	[77]
ChAd3-hliNSmut/MVA-hliNSmut	AdV/MVA	HCV-antigens	/	NCT03688061	1	Completed	[78]
GLS-6150	DNA	NS3/4A/NS4B/NS5A	/	NCT03674125	1	Completed	[79]

### 3.3. HIV

Human immunodeficiency virus (HIV), as a retrovirus, can establish a persistent and stable latent proviral reservoir in quiescent CD4^+^ T cells due to the covalent integration of its single-stranded RNA genome into the host chromosome during infection, which is the fundamental reason for its difficulty in eradication [80,81]. Acquired Immunodeficiency Syndrome (AIDS) thus remains one of the most pressing public health challenges worldwide. Currently, antiretroviral therapy (ART) serves as the standard of care for HIV infection, effectively suppressing viral replication and halting disease progression to AIDS [82]. However, ART fails to eradicate the latent proviral reservoir within the host genome, and viral rebound occurs rapidly in nearly all infected individuals following treatment interruption [12]. Furthermore, long-term antiretroviral therapy (ART) is associated with cumulative toxicities, potential drug resistance risks, as well as psychological and economic burdens [83]. To reprogram and enhance the function of the immune system of people living with HIV (PLWH) through therapeutic vaccines, aiming to induce a broad and potent virus-specific immune response, so as to achieve functional cure without maintenance of ART (long-term virological control), has become the most promising research direction in the field of AIDS research.

Therapeutic HIV vaccines aim to reshape the host immune response by specifically eliminating infected CD4^+^ T cells and neutralizing circulating free virus to block the continuous replication of de novo HIV [84,85]. Induction of potent CD8^+^ T cell responses targeting conserved viral epitopes is a central mechanism for effective immune control [86]. Although the envelope glycoprotein (Env) is still the main target for inducing broadly neutralizing antibodies [87], current strategies focus on targeting highly conserved internal antigens such as Gag, Pol, and Nef to induce cross-reactive T cell immunity [88]. The mRNA-LNP-based protocol to deliver siVmac239-specific antigen has been validated in a non-human primate model and not only amplifies the multifunctional CD8^+^ T cell response, but also promotes reactivation of the latent viral reservoir. This enables “Shock and Kill” functionality on a single platform [89]. This immunization regimen induced a robust Th1-type skewed immune response characterized by markedly increased secretion of IFN-γ, TNF-α, and granzyme B, all of which were strongly associated with delayed viral rebound after treatment interruption. In terms of vaccine vector selection, viral vectors show unique advantages in delivering complex multivalent immunogens. For example, a bifunctional herpes simplex virus vector vaccine encoding SIV-specific Gag, Pol, and Env was demonstrated to simultaneously amplify the magnitude of the multifunctional CD8^+^ T cell response and promote reservoir reactivation in a model of chronic SIV infection, resulting in a significant reduction in the replication-competent viral reservoir. It also significantly delayed viral rebound after antiretroviral therapy interruption (ATI) [90].

The clinical transformation research of AIDS therapeutic vaccines is currently progressing steadily. Most current vaccine candidates, primarily consisting of DNA, viral vector, or dendritic cell platforms encoding specific HIV antigens, have entered clinical testing, although most remain in Phase I or Phase II trials. The HIV clinical studies listed in Table 3 demonstrate that therapeutic vaccines can safely induce HIV-specific T cell responses in humans, but these responses have not consistently translated into durable viral control after antiretroviral therapy (ART) interruption. To date, in randomized controlled trials, no therapeutic vaccine has successfully eradicated the viral reservoir or induced stable ART-free remission. The key limitation is the persistence of latent proviral reservoirs in long-lived memory CD4^+^ T cells and lymphoid tissues, where viral antigen expression is often too low for vaccine-induced effector cells to recognize. In addition, chronic immune activation and progressive T cell dysfunction further reduce the durability of vaccine-induced responses. Therefore, repeated design features in HIV trials include prime–boost vaccination and combination strategies with latency-reversing agents, TLR agonists, broadly neutralizing antibodies, or other immune-modulating approaches. Nevertheless, several vaccines have demonstrated the capacity to safely elicit robust and specific T cell immune responses in early-phase clinical trials, thereby laying a foundation for subsequent optimization strategies.

For example, the PENNVAX-GP DNA vaccine, encoding the Gag, Pol, and Env proteins, when co-administered with the pIL-12 adjuvant, successfully elicited robust HIV-specific T-cell immune responses in HIV-infected participants [91]. Specifically, following the administration of the fourth vaccine dose, the pIL-12-adjuvanted group exhibited a CD4^+^ T cell response rate of up to 96% against HIV Env, Gag, or Pol proteins, while the CD8^+^ T cell response rate ranged from 44% to 64%. AELIX-003, a phase 2a randomized controlled trial, assessed the safety, tolerability, and immunogenicity of a combination regimen comprising HTI immunogen-based vaccines (ChAdOx1.HTI and MVA.HTI) and the TLR7 agonist vesatolimod in 50 early treated, virally suppressed individuals living with HIV-1 [92]. Results demonstrated that the combination regimen was safe and well-tolerated, eliciting robust, broad, and HTI sequence-specific T-cell immune responses. However, during the ATI observation period, no significant differences were observed between the intervention and placebo groups in terms of delaying viral rebound or enhancing viral control.

While the outcomes of completed clinical trials to date remain far from satisfactory, advances in the elucidation of HIV viral structure and infection mechanisms, coupled with the development of immune checkpoint inhibitors, broadly neutralizing antibodies, and latency-reversing agents, have led to an increasing number of clinical trials yielding preliminary positive results. In order to overcome the limitations of single therapy in eliminating the latent viral reservoir, current strategies tend to combine therapeutic vaccines with latent reversal agents, immunomodulators (such as TLR7 agonists, mIL-12, etc.) and other therapeutic methods, and use the “Kick and Kill” strategy to remodel the antiviral immune response [93]. Sequential vaccination regimens, which achieve coverage of multiple HIV-specific proteins via prime-boost strategies and leverage vaccines from diverse technological platforms to elicit complementary immune responses, have also advanced to the early validation phase [94].

**Table 3:** Clinical study of therapeutic vaccines for HIV infection (past decade).

Name	Vaccine Type	HIV Antigens Within Vaccine	Combination Therapy	NCT Number	Phase	Recruitment Status	Ref.
Remune	Inactivated vaccines	gp120	Amplivax	NCT02366026	3	Unknown status	[95]
ChAdOx1.HT and IMVA.HTI	AdV/MVA	HTI	GS-9620	NCT04364035	2	Completed	[96]
PENNVAX-GP	DNA	gag/pol/env	INO-9012 INO-6145	NCT03606213	1/2	Completed	[97]
p24CE1/2 pDNA/ p55gag pDNA	DNA	p24Gag/p55gag	/	NCT03560258	1/2	Completed	[98]
Ad26.Mos.HIV/ MVA-Mosaic	AdV/MVA	gag/pol/env	/	NCT02919306	1/2a	Completed	[99]
MVA HIV-B	MVA	Gag/Pol/Nef	vedolizumab	NCT04120415	2	Completed	[100]
Vacc-4x	peptide	p24	/	NCT01712256	2	Completed	[101]
GTU^®^-Multi HIV B Clade Vaccine	peptide	Rev/Nef/Tat/p17/p24 Protease, RT, gp160 regions of HAN2 HIV-1 B clade.	/	NCT02457689	1/2	Completed	[102]
VAC-3S	Recombinant Proteins	HIV-gp41	/	NCT02390466	1/2	Unknown status	[103]
Ad26.Mos4.HIV MVA-BN-HIV	AdV/MVA	Mos1.Env/Mos2S.Env/Mos1.Gag-Pol/Mos2.Gag-Pol	PGT121	NCT04983030	1/2	Activate Not, Recruiting	[104]
GTU-MultiHIV B-clade vaccine MVA HIV-B HIV vaccine	DNA	Rev/Nef/Tat p17/p24	Vedolizumab	NCT02972450	1/2	Terminated	[105]
THV01-1/THV01-2	LV	Gag/Pol/Nef	/	NCT02054286	1/2	Completed	[106]
ICVAX	DNA	Gag p41-1/Gag p41-2	/	NCT06253533	1	Completed	[107]
DC-HIV04	DC	Inactivated whole autologous HIV/HIV peptides	/	NCT03758625	1	Completed	[108]
HIVARNA01	mRNA	HTI	10-1074 antibodies/ romidepsin	NCT03619278	1	Withdrawn	[109]
HB-502/HB-501	viral vector	Conserved region	/	NCT06430905	1	Terminated	[110]
C62/C1C62/M4/M3M4	AdV/MVA	Gag/Pol/Env/Nef	/	NCT05604209	1	Completed	[111]
D-GPEi/M-GPE	DNA/MVA	Gag/Pol/Env	/	NCT01881581	1	Completed	[112]
GTU-multiHIV/LIPO-5	DNA/peptide	Rev/Nef/Tat p17/p24 Pol/Env	/	NCT01492985	1	Completed	[113]
Tat	Recombinant Proteins	Tat	/	NCT01513135	1	Completed	[114]
Tat Oyi	Recombinant Proteins	Tat	/	NCT01793818	1	Unknown status	[115]

### 3.4. HPV

HPV is a non-enveloped double-stranded circular DNA virus with mucosal tropism, primarily transmitted via sexual contact [116]. The virus infects basal cells through micro-injuries in epithelial tissues and establishes a persistent viral reservoir within the host cell nucleus, thereby driving the progressive development of chronic infection and associated lesions [6]. Persistent infection with high-risk HPV genotypes (e.g., HPV 16 and 18) accounts for the vast majority of cervical cancer cases, as well as cancers at other sites including the oropharynx, anus, and vagina [6]. Current standard clinical interventions mainly include local physical therapies (e.g., laser ablation, cryotherapy) and surgical procedures (e.g., loop electrosurgical excision procedure [LEEP], cold knife conization), with adjuvant radiotherapy and chemotherapy for advanced lesions [117,118]. While these approaches can physically eliminate visible lesions, they fail to eradicate latent viral genomes or integrated DNA fragments in basal cells, resulting in a high risk of post-treatment recurrence [119]. Additionally, existing prophylactic vaccines (e.g., the bivalent Cervarix, quadrivalent Gardasil, and 9-valent Gardasil 9), which induce neutralizing antibodies to block new infections, provide no significant clinical benefit for established chronic infections or precancerous lesions [120]. Given the limitations of current therapies, the development of therapeutic vaccines capable of breaking immune tolerance and clearing viral reservoirs has become an urgent priority. In recent years, breakthroughs in this field have been enabled by in-depth dissection of the molecular mechanisms underlying HPV-induced carcinogenesis, as well as innovations in novel viral vectors, nucleic acid delivery platforms (e.g., DNA, mRNA), and high-efficiency complex adjuvants.

The core of the preclinical research on HPV therapeutic vaccines lies in breaking the immune tolerance of the body to viral oncoproteins and inducing a strong and durable antigen-specific cellular immune response. In terms of antigen selection, the research mainly focuses on the viral oncoproteins E6 and E7, which is quite different from the strategy of preventive vaccines targeting the capsid proteins (L1/L2). E6 and E7 are continuously highly expressed throughout the entire life cycle of HPV infection, especially after the integration of the viral genome, evolving into key drivers for maintaining the malignant phenotype of tumor cells. Therefore, they are ideal targets for immune intervention [121]. These antigenic components are often genetically codon refined or conjugated with immunostimulatory molecules (for example CRT or HSPs) in order to enhance immune stimulation, i.e., to improve antigen handling and cross presentation mechanisms [122,123]. For example, an mRNA vaccine encoding HPV16 E7 delivered via a lipid complex (RNA-LPX) was developed [124]. In animal studies, this vaccine effectively elicited robust antigen-specific CD8^+^ T cell responses, leading to the complete regression of aggressively growing HPV-positive tumors in mice and the establishment of long-term immunological memory. Similarly, the fusion protein vaccine HeDA-HPVE7-16/18 uses TLR4 pathway to precisely target dendritic cells by fusing E7 to human fibronectin superdomain A (hEDA), thereby stimulating potent CTL responses. When combined with cisplatin or Poly ICLC, complete tumor regression was observed in the genital tract tumor model [125]. Recent strategies have also expanded the antigenic repertoire to include E2 and E5 in combinations designed to synergistically enhance the strength and breadth of T cell responses through multiepitope coverage [126,127,128]. In terms of delivery platforms, technical routes are diversified. DNA vaccines are often combined with electroporation technology to improve cellular uptake efficiency, showing good safety and immune persistence. The technical experience accumulated during the COVID-19 pandemic has promoted the mRNA-LNP platform to become the research and development frontier. At the same time, Adv and MVA based vector platforms are still important tools for inducing cellular immunity due to their natural “self-adjuvant” effect and efficient transduction ability.

Due to technological innovation in delivery systems and rapid progress in tumor immunotherapy, the clinical translation of therapeutic HPV vaccines is accelerating. At present, a variety of vaccine candidates have entered clinical evaluation for a broad spectrum of indications, ranging from low-grade intraepithelial neoplasia to recurrent or metastatic malignancies (Table 4). Overall, current HPV therapeutic vaccine studies show more evident clinical potential in early-stage or premalignant lesions, where the immunosuppressive microenvironment is less established and vaccine-induced E6/E7-specific T cell responses may contribute to HPV clearance and histological regression. In advanced HPV-associated cancers, however, the clinical effect of vaccination alone is limited because vaccine-induced T cells must overcome a suppressive tumor microenvironment, including PD-1/PD-L1 signaling, regulatory T cells, and myeloid-derived suppressor cells. Therefore, stronger signals in advanced disease are more often expected from combination strategies with PD-1/PD-L1 inhibitors, other immune checkpoint inhibitors, or tumor microenvironment-modifying therapies. Patient selection according to disease stage, HPV genotype, and tumor immune status is likely to be important for future clinical development. Against this background, different vaccine platforms have been evaluated in clinical studies.

In terms of nucleic acid vaccine platforms, DNA vaccines significantly improve the quality of cellular immune responses by virtue of electroporation technology. In the case of VGX-3100, which targets the HPV-16/18 E6/E7 oncoprotein and is delivered by the CELLECTRA electroporation system, the vaccine successfully achieved significant histologic regression and viral clearance in a clinical trial of high-grade cervical squamous intraepithelial lesion (CIN2/3) [129]. Another DNA vaccine, GX-188E, enhances the recruitment and activation of dendritic cells by fusing the Flt3L sequence. It has been confirmed in the Phase II study that it has the ability to induce E6/E7-specific T cells. Moreover, when used in combination with the PD-1 monoclonal antibody (pembrolizumab) for the treatment of recurrent or advanced cervical cancer, it has demonstrated an encouraging synergistic anti-tumor effect [130]. Meanwhile, the mRNA-LNP platform also shows great potential. For example, BNT113, encoding HPV-16 E6/E7, is currently in phase II clinical trials to evaluate its translational efficacy in combination with immune checkpoint inhibitors in HPV-positive head and neck squamous cell carcinoma [131]. In terms of viral vector platforms, they have become a research hotspot due to their efficient T cell priming ability. TG4001, a modified vaccinia Ankara virus (MVA)-based vaccine, by co-expressing IL-2 as a molecular adjuvant, in combination with an anti-PD-L1 agent (avelumab) has shown clear signs of tumor regression and robust immunogenicity in HPV-16-positive recurrent/metastatic cancers in a phase Ib/II trial [132]. Another notable candidate, PRGN-2012, which uses a gorilla adenovirus vector to express HPV-16/11 antigens, demonstrated excellent safety in a Phase I clinical trial for adult patients with recurrent respiratory papillomatosis (RRP), significantly inducing HPV-specific T cells and significantly reducing the patients’ reliance on subsequent surgical procedures [133]. To further overcome vector immunity, the candidate drugs HB-201 and HB-202 innovatively employ a heterologous prime-boost strategy using arenavirus vectors and have shown excellent potential for immune activation in early trials targeting HPV-16 positive cancers [134].

Clinical evidence indicates that although single-agent therapeutic HPV vaccines are effective in inducing antigen-specific immune responses, their clinical efficacy is significantly dependent on the course of disease, and it is difficult to completely overcome the highly immunosuppressive microenvironment (TME) in advanced malignancies [135]. In the early stages of the disease, such as low-grade cervical intraepithelial neoplasia, a single vaccine intervention can bring significant clinical benefit because systemic immunosuppression has not yet been fully established. For example, the VTP-200 vaccine, which uses a heterologous prime-boost strategy of chimpanzee adenovirus (ChAdOx1) and modified MVA to target the expression of HPV early proteins, successfully induced robust and specific T-cell responses and showed encouraging viral clearance in patients with early high-risk HPV infection [136]. However, once precancerous lesions progress to invasive carcinomas, newly generated T cells frequently undergo rapid exhaustion upon entering the PD-L1-rich and Treg-infiltrated local TME. Consequently, rational combination therapies have become a critical strategy in vaccine development. In current clinical studies, PD-1/PD-L1 inhibitors are often used together with vaccines to combat this immunosuppressive tumor microenvironment. This intervention was designed to block the transition of cytotoxic T cells that would inhibit vaccine activation, thereby allowing these activated cytotoxic T cells to persist for a longer period to better kill the tumor. For example, recent Phase II clinical data revealed that pairing the APC-targeted DNA vaccine VB10.16 with the PD-L1 inhibitor atezolizumab achieved a remarkable objective response rate and significantly extended overall survival in patients with advanced, previously treated HPV16-positive cervical cancer [137]. Similarly, the combination of the nanoparticle-based vaccine PDS0101 and pembrolizumab has yielded highly encouraging clinical responses and durable T cell infiltration in patients with recurrent or metastatic HPV16-positive head and neck squamous cell carcinoma, further validating the necessity of disrupting the TME to unlock the full potential of vaccine-elicited immunity [138].

**Table 4:** Clinical study of therapeutic vaccines for HPV infection (past decade).

Name	Vaccine Type	HPV Antigens Within Vaccine	Combination Therapy	Indication	NCT Number	Phase	Recruitment Status	Ref.
VGX 3100	DNA plasmids	E6/E7	/	HSIL	NCT03185013	3	Completed	[139]
			/	HSIL	NCT03721978	3	Completed	[140]
ADXS11-001	Bacterial vector	E7	/	HRLACC	NCT02853604	3	Terminated	[141]
BNT-113	mRNA	E6/E7	Pembrolizumab	HNC	NCT04534205	2/3	Recruiting	[131]
			/	HNC	NCT03418480	1/2	Completed	[142]
HB-200 (HB-201/HB-202)	LCMV/PICV	E6/E7	Pembrolizumab	HNC	NCT06513884	2/3	Withdrawn	[143]
			/	HNC	NCT04180215	1/2	Completed	[144]
			/	HNSCC	NCT06373380	1	Activate Not, Recruiting	[145]
GX-188E	DNA plasmids	E6/E7	GX-I7 nivolumab	HNSCC	NCT05280457	2	Recruiting	[146]
			GX-I7 Pembrolizumab	HNC	NCT05286060	2	Activate Not, Recruiting	[147]
PRGN-2009	GC46	E5/E6/E7	Pembrolizumab	OPSCC	NCT05996523	2	Activate Not, Recruiting	[148]
Vvax001	SFV	E6/E7	/	HSIL	NCT06015854	2	Unknown status	[149]
			/	HSIL	NCT03141463	1	Completed	[150]
ISA101b	Peptide	E6/E7	Cemiplimab	OPSCC	NCT04398524	2	Unknown status	[151]
			Cemiplimab	OPSCC	NCT03669718	2	Unknown status	[152]
			Utomilumab	HNC	NCT03258008	2	Terminated	[153]
PDS0101	Peptide	E6/E7	Pembrolizumab	HNSCC	NCT04260126	2	Completed	[154]
			Cisplatin	LACC	NCT04580771	2	Activate Not, Recruiting	[155]
PVX-2	DNA plasmids/ Fusion protein	E6/E7/L2	/	SIL	NCT03911076	2	Terminated	[156]
VB10.16	DNA plasmids	E6/E7	Atezolizumab	HPV 16-positive Cervical Cancer	NCT04405349	2	Completed	[157]
			/	HSIL	NCT02529930	1/2	Completed	[158]
			Pembrolizumab	HNC	NCT06016920	1/2	Recruiting	[159]
TA-HPV	DNA plasmids	E6/E7	Pembrolizumab	OPSCC	NCT05799144	2	Recruiting	[160]
MEDI0457	DNA plasmids	E6/E7	IL-12 DNA plasmids	HPV associated cancers	NCT03439085	2	Terminated (Project delays)	[161]
PepCan	Peptide	E6	Candin^®^	HSIL	NCT02481414	2	Completed	[162]
			/	HNC	NCT03821272	1/2	Completed	[163]
BVAC-C	Autologous B cells and monocytes	E6/E7	Topotecan	Cervical Cancer	NCT02866006	1/2	Completed	[164]
TG-4001	MVA	E6/E7	Avelumab	Recurrent or Metastatic Malignancies	NCT03260023	1/2	Activate Not, Recruiting	[165]
PRGN-2012	GC46	E6/E7 (HPV-6/11)	/	RRP	NCT04724980	1/2	Activate Not, Recruiting	[166]
VTP-200	AdV/MVA	E1/E2/E4/E5/E6/E7	/	LSIL	NCT04607850	1/2	Completed	[167]
DPX-E7	Peptide	E7	CTX	Oropharyngeal cancer cervical cancer anal cancer	NCT02865135	1/2	Completed	[168]
MVA.HPV16/18 Ad26.HPV16	AdV/MVA	E2/E6/E7	/	HPV-associated cervical infection	NCT03610581	1	Terminated	[169]

### 3.5. EBV

Epstein–Barr virus (EBV), as a widely spread γ-herpesvirus among the global population, possesses a large double-stranded DNA genome and has a unique lymphotropic affinity for B lymphocytes and epithelial cells [170]. The primary infection usually begins in the oropharyngeal mucosa, and the virus then persists through a lifelong latent infection in the form of an Episome in the host nucleus [171]. Although in hosts with normal immune function, this persistent infection usually presents as an asymptomatic viral carrier state, the latent viral reservoir is a key oncogenic driver for various malignant tumors, including nasopharyngeal carcinoma (NPC), gastric cancer, Burkitt lymphoma, and Hodgkin lymphoma, etc. [172]. Current clinical interventions mainly rely on broad-spectrum radiotherapy and chemotherapy, often accompanied by severe systemic toxicity. However, traditional antiviral drugs, such as acyclovir or ganciclovir, only target the lytic replication phase of the virus and are completely ineffective against the latent reservoir of infection that maintains the malignant phenotype [173]. Since there is no prophylactic vaccine that can effectively block the primary infection, the development of therapeutic vaccines that can overcome the immune escape mechanism of EBV and specifically eliminate malignant cells carrying latent antigens has become an urgent clinical need.

Central to the preclinical development of therapeutic EBV vaccines is deciphering viral latency and reshaping T cell immune surveillance against infected cells. Unlike prophylactic vaccines that target gp350, a lytic glycoprotein, to induce neutralizing antibodies, the antigenic design of therapeutic vaccines focuses on latent period-specific proteins, mainly EBV nuclear antigen 1 (EBNA1) and latent membrane protein 1 and 2 (LMP1/LMP2). The persistent expression of these antigens in EBV-associated malignancies, such as nasopharyngeal carcinoma, lymphoma, is a key driver in maintaining the malignant phenotype of tumor cells [174]. However, the natural latent antigens have significant immunogenicity defects and must be rationally designed to ensure safety and efficacy. For EBNA1, its specific glycine-alanine (Gly-Ala) repeating sequence hinders proteasome processing, preventing the protein from being presented via MHC I class molecules to CD8^+^ T cells. Therefore, modern vaccines often use truncated mutants (such as EBNA1ΔGA), by deleting this inhibitory domain to restore the antigen presentation efficiency. For LMP1, due to its constitutively activated oncogenic activity similar to CD40, vaccine design requires targeted deletion or inactivation of its signaling domain to achieve “functional attenuation” without losing immunogenicity [174,175]. In terms of delivery strategies, multiple innovative platforms are striving to overcome host immune tolerance. In the context of viral vector platforms, the modified Ankara vaccinia virus (MVA) vector (such as MVA-EL) can simultaneously deliver the optimized EBNA1ΔGA and LMP2 antigens, effectively infect antigen-presenting cells, thereby inducing a multifunctional, Th1-polarized CD4^+^ T cell response and CD8^+^ T cells with strong tumor-killing activity, and achieving rapid clearance of EBV-positive tumors in the model [176]. In terms of nano and peptide platforms, synthetic extended peptide chains (SLPs) or nanoparticles loaded with LMP2 epitopes, when combined with pattern recognition receptor agonists such as CpG oligodeoxynucleotides or Poly I:C, can significantly enhance dendritic cell maturation and local interferon-mediated immune responses. These optimized preclinical systems lay a solid mechanistic foundation for reversing the immunosuppressive state of the tumor microenvironment and ultimately achieving clinical transformation.

With the increasing maturity of antigen optimization strategies, the clinical translation of EBV therapeutic vaccines is accelerating. Currently, a multi-pathway clinical development strategy involving viral vectors, dendritic cell (DC)-based vaccines, and nucleic acid vaccines has been established (Table 5). The EBV studies listed in Table 5 suggest that vaccines targeting latent antigens such as EBNA1 and LMP2 can induce EBV-specific T cell responses, but the translation of these responses into durable clinical benefit remains limited. This is largely because EBV persists in latent cellular reservoirs, mainly B cells and epithelial cells, and EBV-associated tumors can further impair antigen presentation and establish an inhibitory tumor microenvironment. Compared with non-oncogenic chronic viral infections, EBV-associated diseases often require tumor-oriented endpoints, such as EBV DNA reduction, tumor response, delayed progression, or survival benefit.

Among the current vaccine platforms, viral vector-based approaches have been widely explored because of their inherent adjuvant effect and efficient T cell priming ability. Mva-el is a typical vaccine, which uses the modified vaccinia Ankara virus (MVA) backbone to express a fusion protein consisting of a truncated (C-terminal) EBNA1 and full-length LMP2. In a phase I/II clinical study of EBV-associated NPC, the vaccine successfully induced robust helper and cytotoxic T cell responses that were not restricted by the HLA polymorphism of the patients, demonstrating broad therapeutic potential [177]. In the field of cell vaccines, the vaccine strategy based on autologous DC loaded with LMP1/LMP2 peptide segments has achieved proof-of-concept in phase II clinical trials. The data show that this therapy not only effectively stimulates specific CTL responses targeting LMP proteins, but also, during the follow-up period, patients receiving treatment showed significantly delayed tumor progression compared to the control group [178]. Moreover, the nucleic acid vaccine platform has emerged as a standout option due to its advantages of rapid production and multi-epitope coverage. The new delivery system based on plasmid DNA and mRNA-LNP aims to prevent the recurrence of EBV-related lymphomas by expressing multiple epitopes of EBV latent antigens. It is currently in the early stage of clinical evaluation, aiming to verify its potential in maintenance therapy [178].

Although therapeutic vaccines can induce specific T cell responses, clinical evidence indicates that in advanced EBV-related malignant tumors, a single vaccine intervention often fails to overcome the highly immunosuppressive tumor microenvironment (TME). Taking nasopharyngeal carcinoma (NPC) as an example, its TME is rich in Tregs and myeloid-derived suppressor cells (MDSCs) and is accompanied by high expression of virus-driven PD-L1, jointly forming a powerful immunosuppressive barrier [179]. This makes the EBNA1/LMP2-targeting effector T cells highly vulnerable to exhaustion and inactivation after entry into the tumor site, even if the vaccine successfully activates them. To meet this challenge, a combination therapy strategy has become the core direction of clinical translation. Current research focuses on combining vaccines with immune checkpoint inhibitors, especially blocking the PD-1/PD-L1 pathway. This synergistic mechanism can effectively release the inhibitory “brake” on vaccine-induced CTLs and maintain their survival ability and killing activity in TME. In addition, therapeutic vaccines are beginning to be combined with adoptive T cell therapy (ACT), such as ex vivo expanded EBV-specific CTLs. In this model, the vaccine not only acts as an immunome, but also acts as an “in vivo amplificant” to promote the persistent survival and function of the adoptively transferred T cells in vivo, thereby achieving the synergistic control of refractory EBV-associated tumors.

**Table 5:** Clinical study of therapeutic vaccines for EBV infection (past decade).

Name	Vaccine Type	EBV Antigens Within Vaccine	Combination Therapy	NCT Number	Phase	Recruitment Status	Ref.
MVA-EBNA1/LMP2	MVA	EBNA1/LMP2	/	NCT01094405	2	Completed	[180]
MVA-EL	MVA	EBNA1/LMP2	/	NCT01147991	1	Completed	[181]
Ad5-EBV-LMP2	AdV	LMP2	/	NCT00078494	1	Completed	[182]
LPX-mLMP2	mRNA	LMP2	/	NCT05714748	1	Activate Not, Recruiting	[183]
mRNA-1195	mRNA	H, gL, gp42, gp220/gp350, gB	/	NCT05831111	1	Activate Not, Recruiting	[184]
WGc-043	mRNA	/	/	NCT07028047	1	Not yet recruiting	[185]

## 4. Key Challenges and Future Perspectives

### 4.1. Challenges in Clinical Translation

**Figure 2:**
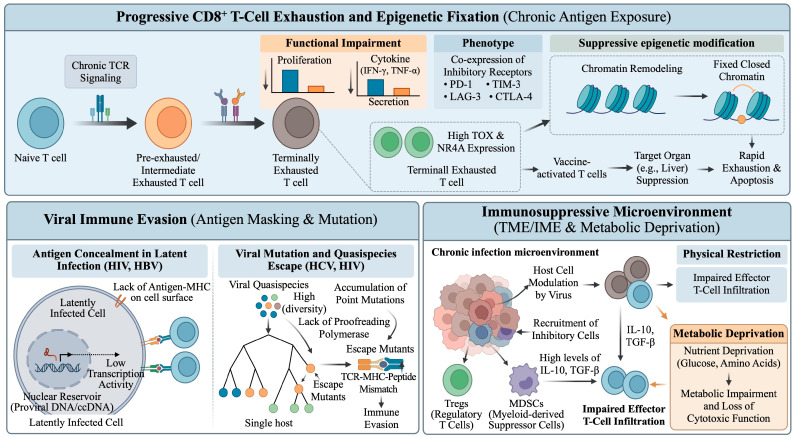
Major mechanisms for the limited efficacy of therapeutic vaccines in chronic viral Infection. Therapeutic vaccine efficacy is limited by persistent viral reservoirs, reduced antigen visibility, chronic antigen-driven T cell exhaustion, viral immune escape, impaired antigen presentation, and suppressive tissue or tumor microenvironments. These barriers differ among HBV, HCV, HIV, HPV, and EBV infections, but collectively explain why vaccine-induced immune responses often fail to achieve durable viral control, lesion regression, tumor response, or functional cure.

The above preclinical and clinical studies of therapeutic vaccines for chronic viral infections show that multiple candidate therapeutic vaccines can induce virus-specific immune responses in animal models and early-phase clinical trials. In preclinical models, these candidates often generate favorable immunological signals, including activation of antigen-specific T cells, enhancement of Th1-type immune responses, reduction of viral biomarkers, or inhibition of virus-associated tumors. However, these positive immune responses do not always translate into durable virological suppression, lesion regression, tumor control, or functional cure in patients. Clinical endpoints are often only partially achieved or are transient. For example, in clinical trials without concomitant antiviral drugs or other therapeutic interventions, vaccines usually fail to achieve sustained viral suppression, reduction of viral antigen burden, clearance of precancerous lesions, or long-term control or remission of associated malignancies. As shown in Figure 2, an important reason for this limited clinical efficacy is the complex immunosuppressive network established during chronic infection, including viral reservoirs, insufficient antigen visibility, T cell exhaustion, viral immune escape, and suppressive local tissue microenvironments.

The immunosuppressive microenvironment is closely related to the cellular or tissue compartments that support persistent infection by different viruses. The immunological constraints formed by viral reservoir characteristics, antigen accessibility or visibility, and the local immune state are important factors affecting the clinical efficacy of therapeutic vaccines.

During HBV infection, nuclear cccDNA persists as a chromatinized minichromosome within hepatocytes. The transcriptional activity of this minichromosome is not static but is strictly controlled by histone modifications. While active marks promote viral gene expression, repressive marks such as H3K9me3 (deposited by SUV39H1/SETDB1) and H3K27me3 (mediated by PRC2/EZH2) enforce transcriptional silencing. Consequently, cccDNA can be maintained at low intrahepatic copy numbers for extended periods, thereby undermining efforts to achieve a functional cure [186,187]. The liver has an intrinsic tendency toward immune tolerance [188]. Intrahepatic Kupffer cells, liver sinusoidal endothelial cells (LSECs), and regulatory T cells (Tregs) collectively shape an immunosuppressive/tolerogenic milieu—mediated by PD-L1 expression on non-parenchymal cells and by suppressive cytokines (IL-10, TGF-β)—which promotes functional exhaustion of HBV-specific CD8^+^ T cells through co-inhibitory pathways including PD-1/PD-L1 and, potentially, BTLA–HVEM. Consequently, vaccine-elicited peripheral effector T cells may still face a constrained fitness landscape upon entry into the liver, limiting durable clearance of cccDNA-harboring hepatocytes [189,190,191].

HCV generates high-mutational-rate quasispecies because its RNA-dependent RNA polymerase lacks proofreading activity, thereby exposing vaccine-induced antibody and T cell responses to immune escape pressure [192]. In addition, the HCV NS3/4A protease cleaves mitochondrial antiviral signaling protein (MAVS) and the TLR3 adaptor protein TRIF, thereby blocking RIG-I-like receptor and TLR3-mediated type I interferon induction pathways, weakening type I interferon production and downstream interferon-stimulated gene (ISG) induction, and consequently limiting the ability of therapeutic vaccines to restore local antiviral immune responses [193,194].

In HIV infection, replication-competent (or intact) proviral DNA is stably integrated into the genome of resting memory CD4^+^ T cells, with central memory (Tcm) and transitional memory (Ttm) subsets representing key components of the long-lived latent reservoir [195,196]. Many of these proviruses reside in a transcriptionally silent or minimally active state, such that viral antigen expression can be absent or kept at extremely low levels and is often insufficient for robust immune recognition [197]. In addition, the B cell follicle/germinal center within lymphoid tissues functions as an anatomical compartment where CD8^+^ effector T cells are relatively excluded (e.g., limited CXCR5-mediated follicular access) [198,199]; this spatial restriction further constrains vaccine-induced immune surveillance from effectively covering regions enriched in latently infected cells.

In the basal/proliferating layer, viral episodes are maintained with low-level early gene expression (including E1/E2 and restrained E6/E7), whereas the more immunogenic late capsid proteins L1/L2 are largely confined to terminally differentiated superficial cells and are shed with desquamation, limiting effective antigen exposure to the underlying immune system [200,201]. HR-HPV oncoproteins further remodel the epithelial immune landscape: E5 and E6/E7 impair MHC-I–restricted antigen presentation by downregulating/repressing MHC-I heavy chain, TAP1 and LMP2, and by disrupting innate sensing/inflammatory signaling (e.g., NF-κB–dependent outputs), thereby damping local IFN/chemokine milieux that recruit and activate effector leukocytes [202,203,204]. These events, together with lesion-associated increases in immunosuppressive cytokines IL-10/TGF-β and accumulation of Tregs and MDSCs, consolidate an immunosuppressive micromilieu that is difficult for therapeutic vaccines alone to reverse and eliminate infected keratinocytes [205].

During EBV latency, the N-terminal glycine-alanine repeat (GAr) domain of EBNA1 suppresses translation initiation of its own mRNA (via nucleolin recruitment to GAr-encoding RNA G4 structures), limiting full-length EBNA1/peptide supply for MHC-I-restricted presentation [206,207]. Concurrently, EBNA2 represses the MHC-II transactivator CIITA through an enhancer-switching mechanism at the CIITA-DEXI locus, lowering HLA-II expression [208,209], while BART miRNAs further erode both classical (TAP-dependent MHC-I loading) and innate recognition pathways (e.g., MICA/NKG2D) [170,210]. This multilayered interference with antigen processing/presentation can blunt vaccine-elicited CTL recognition of latently infected or transformed cells.

Among these virus-specific barriers, T cell exhaustion represents a common mechanism linking persistent antigen exposure to the limited efficacy of therapeutic vaccination. Unlike prophylactic vaccines, therapeutic vaccines are administered after viral antigens have persisted in the host for a prolonged period. Under these conditions, virus-specific CD8^+^ T cells are repeatedly stimulated through the T cell receptor (TCR), and this sustained TCR signaling is a central driver of the T cell exhaustion program [10]. This process should not be understood simply as “T cell fatigue”; rather, chronic antigen stimulation gradually reshapes the differentiation state of virus-specific T cells through transcriptional and epigenetic mechanisms [211].

At the molecular level, the calcineurin-NFAT pathway serves as an important hub connecting sustained TCR stimulation with the T cell exhaustion program [212,213]. During acute immune responses, NFAT cooperates with AP-1 to promote effector molecule expression and cytotoxic differentiation. In contrast, under chronic antigen stimulation, prolonged NFAT activity or NFAT activity insufficiently coupled to AP-1 is more likely to induce exhaustion-associated transcriptional programs [214]. Existing studies have shown that NFAT can induce TOX/TOX2 and members of the NR4A transcription factor family, which participate in the establishment and maintenance of CD8^+^ T cell exhaustion [215]. Therefore, molecules such as PD-1, TIM-3, LAG-3, TIGIT, and CTLA-4 should not be regarded merely as surface markers of exhausted T cells, but rather as phenotypic manifestations of an exhaustion-associated transcriptional program driven by chronic antigen stimulation [214]. Further studies indicate that exhausted T cells are not a homogeneous population. Under chronic stimulation, virus-specific CD8^+^ T cells can give rise to TCF1^+^ progenitor-like exhausted T cells and more differentiated TCF1^−^ terminally exhausted T cells. The former retains a degree of self-renewal and proliferative capacity and can generate downstream effector-like exhausted progeny. They represent an important cellular source of proliferative responses after PD-1/PD-L1 blockade and may also constitute the T cell pool most likely to be mobilized by therapeutic vaccination. In contrast, terminally exhausted T cells usually express high levels of multiple inhibitory receptors, show reduced cytokine production and proliferative capacity, and have limited ability to re-establish stable effector- or memory-like states [216,217,218].

The limited reversibility of terminal exhaustion is closely associated with its stable epigenetic state [219]. Exhausted CD8^+^ T cells display a chromatin accessibility landscape distinct from that of effector and memory T cells, and exhaustion-associated genes and regulatory regions, such as PDCD1, HAVCR2, LAG3, and ENTPD1, can remain persistently accessible [214]. Even when the PD-1/PD-L1 pathway is blocked, cytokine production, proliferation, or short-term cytotoxic function may be only partially restored, without fully resetting terminally exhausted T cells to a classical memory T cell state [220]. This may explain why some therapeutic vaccine studies detect increased numbers of virus-specific T cells or enhanced IFN-γ responses in peripheral blood, whereas such immunogenicity does not necessarily translate into sustained viral suppression or reservoir clearance [211]. Therefore, when viral antigens continue to be produced, simply increasing vaccine immunogenicity or administering frequent booster doses may have limited benefit. After newly expanded virus-specific T cells enter target tissues, they may still encounter persistent TCR stimulation, co-inhibitory signaling, and a local immunosuppressive environment, which can drive them back into an exhausted state. For chronic infections with high antigen burden, reducing antigen input first through direct antiviral therapy, siRNA, antigen-lowering strategies, or inhibition of viral transcription may help decrease sustained TCR stimulation. Therapeutic vaccination may then expand T cell pools that still retain responsiveness, while checkpoint blockade or other immunomodulatory strategies applied within an appropriate window may help preserve their effector function. Thus, the sequential design of antigen reduction, vaccine boosting, and checkpoint modulation may be as important as the magnitude of vaccine-induced immune responses.

Another important challenge is that the intended meaning of “functional cure” differs across viral diseases. As shown in Table 6, for HBV and HIV, functional cure generally refers to durable viral control after discontinuation of antiviral therapy. In HCV infection, because DAAs already achieve high sustained virological response rates, therapeutic vaccines may be more relevant for preventing reinfection or enhancing durable immune protection. For HPV and EBV-associated diseases, clinically meaningful endpoints usually include lesion regression, viral clearance, tumor response, delayed disease progression, or survival benefit, rather than complete elimination of all viral genomes.

These disease-specific differences in endpoints not only affect the interpretation of efficacy, but also directly influence trial duration, sample size estimation, follow-up strategies, and cost-effectiveness assessment. Current standard therapies for HBV and HIV, such as nucleos(t)ide analogues and ART, are widely used in clinical practice and can continuously suppress viral replication with controllable costs and manageable adverse effects. Therefore, the development of novel therapeutic vaccines usually needs to demonstrate clear clinical advantages over existing therapies. Given the long development cycle and high investment required for vaccine development, limited improvements in final efficacy may lead to uncertainty in return-on-investment (ROI) assessments. In addition, evaluation of the clinical endpoint of “functional cure” usually requires long-term follow-up. In chronic HBV infection, for example, clinical evaluation criteria generally require participants to maintain HBsAg loss and undetectable HBV DNA for a defined period after discontinuing antiviral therapy, such as 6 months or longer, to confirm durable immune control [225]. These endpoint definitions are intended to ensure patient safety and verify the long-term efficacy of the intervention. However, in clinical trials, prolonged off-treatment follow-up inevitably extends the study duration, increases resource requirements, and requires continuous monitoring for potential viral rebound.

Moreover, the complexity of clinical trial design and the limitations of early evaluation indicators further increase the difficulty of development. Under the current trend toward combination therapy, it is methodologically challenging to accurately evaluate the individual efficacy and safety contributions of each vaccine-related component. This often requires multi-arm trials, thereby increasing the required sample size. At the same time, because fully validated early surrogate biomarkers that can reliably predict long-term cure endpoints are lacking, developers have difficulty making early go/no-go decisions during trials. They usually have to rely on long-term follow-up to confirm final efficacy, which objectively prolongs clinical development. Most currently available standard antiviral drugs are oral formulations that can be stored at room temperature, facilitating patient adherence and distribution in primary care settings. In contrast, some novel vaccine platforms, such as mRNA vaccines requiring specific cold-chain transportation or DNA vaccines requiring electroporation devices, involve more complex storage conditions and administration procedures. These logistical requirements may affect accessibility in resource-limited regions, which are often areas with a high burden of chronic viral infections.

### 4.2. Future Perspectives

Based on the above immunological and clinical translational barriers, future development of therapeutic vaccines should shift from simply enhancing immunogenicity toward designing verifiable, integrated intervention strategies according to the dominant bottlenecks of each disease. In other words, vaccine platforms, combination partners, dosing sequences, and clinical endpoints should all serve a central goal: enabling vaccine-induced immune responses to achieve sustained viral control or lesion clearance within target tissues.

This concept is first reflected in the development of sequential combination therapy. For diseases with persistently high antigen burden, such as chronic HBV infection, antigen-lowering strategies, including antiviral agents, siRNA, antisense oligonucleotides, or neutralizing antibodies, may reduce sustained TCR stimulation before or during vaccination, thereby creating conditions for the functional recovery of virus-specific T cells [226,227,228,229,230]. For diseases characterized by latent reservoirs or insufficient antigen visibility, such as HIV and EBV infection, therapeutic vaccines may need to be combined with latency reversal, enhanced antigen presentation, or viral reservoir-targeting strategies to increase the recognizability of target cells by effector T cells [86,224,231,232]. On this basis, immune checkpoint blockade or other immunomodulatory approaches may help maintain the effector function of vaccine-induced T cells in target tissues. Therefore, the timing and sequence of antigen reduction, vaccine boosting, and immune modulation may influence final efficacy as much as the vaccine platform itself.

Antigen design should also move beyond the sole pursuit of immunogenicity and instead incorporate antigen conservation, disease relevance, and accessibility to MHC presentation. For highly variable viruses such as HCV and HIV, candidate antigens should preferentially cover evolutionarily conserved and functionally constrained regions to reduce the risk of epitope escape [65,86,233]. For HBV, HPV, and EBV, antigen selection should also consider the actual expression level and MHC presentation of candidate antigens in reservoir cells, chronically infected tissues, or virus-associated tumor cells [234,235]. Multi-omics analysis, Immunopeptidomics, structural prediction, and artificial intelligence-assisted reverse vaccinology can be used to identify conserved epitopes, optimize multi-antigen combinations, and predict MHC-binding capacity [236,237,238,239]. However, computational prediction should only serve as a tool for prioritizing candidate antigens; their presentation, immunogenicity, and disease relevance still require validation in in vitro immunological assays, animal models, and clinical samples.

Optimization of delivery systems and immunization regimens should serve the establishment of effective immune responses within target tissues. mRNA-LNP platforms, DNA vaccines delivered by electroporation, viral vectors, and heterologous prime–boost strategies can all enhance cellular immunity; however, their translational value should not be judged solely by the magnitude of immunogenicity in peripheral blood. Future platform evaluation should further consider the duration of antigen expression, the type of antigen-presenting cells involved, the tissue-homing capacity of T cells, intralesional infiltration, and maintenance of local effector function [240,241]. For diseases in which CD8^+^ T cell-mediated clearance is central, promotion of MHC-I presentation and cross-presentation remains an important goal for delivery system optimization. For diseases constrained by liver tolerance or the tumor microenvironment, local immunomodulatory strategies should be incorporated to maintain the effector function of vaccine-induced T cells within lesions.

In addition to antigen delivery and the efficiency of T cell priming, the innate immune environment also influences whether therapeutic vaccine-induced responses can be maintained at disease sites. Although therapeutic vaccines primarily aim to restore or enhance virus-specific adaptive immunity, especially CD8^+^ T cell-mediated cytotoxic responses and CD4^+^ T cell help, their efficacy does not depend solely on adaptive immune cells. The innate immune system substantially influences the quality of antigen presentation, the intensity of inflammatory signaling, the tissue chemokine milieu, and the local antiviral state, all of which are important prerequisites for the effective establishment of vaccine-induced effector responses at lesion sites [242,243]. DC cell maturation and cross-presentation capacity, type I/type III interferon signaling, early recognition by NK cells, inflammatory regulation by monocytes/macrophages, and the intrinsic antiviral state of epithelial cells may all affect the expansion, migration, and local functional maintenance of vaccine-induced T cells. Therefore, future therapeutic vaccine design should not focus only on antigen selection and the magnitude of adaptive immune responses, but should also consider how adjuvants, delivery systems, and combined immunomodulatory strategies can reshape the innate immune environment to provide appropriate priming and effector conditions for subsequent adaptive immunity.

Genital herpes caused by HSV provides an additional perspective for understanding the relationship among local innate immunity, tissue-resident immunity, and therapeutic vaccine efficacy. HSV establishes latency in sensory ganglia, whereas recurrent lesions and viral shedding mainly occur in peripheral skin or mucosal tissues [244]. Because viral antigen expression in latently infected neurons is limited, therapeutic vaccines may have limited capacity to directly eliminate latent reservoirs solely through circulating T cell responses. Instead, their potential value may lie in restricting local viral expansion after reactivation, shortening the duration of viral shedding, and reducing recurrence frequency. This process is highly dependent on local immune control during the early phase of viral reactivation, including interferon responses in mucosal epithelial cells, rapid recognition by dendritic cells and NK cells, regulation of inflammatory magnitude by monocytes/macrophages, and immediate local effector activity mediated by tissue-resident memory T cells [245,246]. Therefore, for HSV and other latent infections with recurrent reactivation, future evaluation endpoints for therapeutic vaccines should not be limited to peripheral blood T cell responses, but should also include viral shedding, recurrence frequency, local mucosal innate immune status, and tissue-resident immune function.

In addition, clinical trial design should move beyond simple evaluation of platform safety and immunogenicity toward mechanism-driven efficacy assessment and patient stratification. Patients differ substantially in baseline antigen burden, reservoir activity, degree of T cell exhaustion, disease stage, and local immune status, all of which may affect the efficacy of therapeutic vaccines [224,226]. Future studies should prioritize stratification strategies based on viral antigen levels, reservoir-related markers, T cell functional status, and tissue immune features. In combination therapy trials, multi-arm or adaptive designs should be used when appropriate to distinguish the contributions of the vaccine, antigen-lowering agents, and immunomodulators. Early surrogate markers should also expand beyond single IFN-γ responses to include more mechanism-informative indicators, such as virus-specific T cell polyfunctionality, TCR clonal expansion, changes in exhaustion markers, tissue infiltration, and early reduction of viral antigens.

The clinical value of therapeutic vaccines should also be evaluated in the context of disease-specific endpoints and real-world accessibility. For HBV, greater emphasis should be placed on sustained HBsAg loss, long-term HBV DNA control, and maintenance of response after treatment discontinuation. For HIV, key endpoints include delayed viral rebound after ART interruption or ART-free viral control. In the era of DAAs, the value of HCV vaccines may mainly lie in preventing reinfection or improving long-term immune protection in high-risk populations. For HPV- and EBV-associated diseases, translational evaluation should be based on endpoints such as lesion regression, decline in viral DNA, tumor remission, progression-free survival, or overall survival. At the same time, mRNA-LNP cold-chain requirements, electroporation devices for DNA vaccines, repeated immunization regimens, and complex combination therapies may all affect clinical implementation. Improving the thermal stability of mRNA-LNP formulations through lyophilization and other formulation engineering approaches, simplifying administration procedures, and reducing the complexity of combination regimens are important conditions for advancing therapeutic vaccines from early clinical research toward practical application.

## 5. Conclusions

Therapeutic vaccine is a key strategy to achieve functional cure of chronic infections such as hepatitis B virus (HBV), hepatitis C virus (HCV), human papillomavirus (HPV), human immunodeficiency virus (HIV), human papillomavirus (EBV), and so on. Although significant breakthroughs have been made in antigen design and delivery systems, their clinical translation still faces severe challenges. The core obstacle lies in the deep immunosuppressive network established by chronic infection.

To break through the above limitations, clinical development has shifted from a single vaccine therapy to a multi-modal combined strategy. The ideal intervention requires a synergistic effect on the viral infection mechanism and the host immune pathway. By using direct antiviral drugs or antigen reduction techniques to reduce viral load and alleviate the continuous stimulation of T cells, combined with immune checkpoint inhibitors (such as PD-1/PD-L1 blockers) to relieve the suppression of the immune microenvironment, the vitality and cytotoxicity of effector T cells induced by vaccines can be maintained. In the future, the next generation of therapeutic vaccines will rely on artificial intelligence and reverse vaccinology technology to achieve accurate multi-epitope antigen selection and immunogen optimization, overcoming the limitations of traditional empirical design. Combined with the continuous improvement of formulation technology to enhance stability and delivery efficiency, these comprehensive strategies will provide a scientifically feasible path to overcome current immune barriers and restore persistent antiviral immunity, ultimately becoming an important approach for treating chronic viral infections.

## Figures and Tables

**Table 6:** Functional cure or clinically relevant endpoints of therapeutic vaccines for different chronic infectious diseases.

Infection	Functional Cure or Practical Endpoint	Evidence from Cited Vaccine Studies
HBV	sustained HBsAg loss and HBV DNA less than the lower limit of quantitation (LLOQ) 24 weeks off treatment [221]	Rare; partial HBsAg reduction and immune responses observed, especially in combination regimens
HCV	SVR is already achieved by DAAs; vaccine endpoints may include prevention of reinfection or durable immune protection in selected populations [222,223].	Mostly immunogenicity; limited clinical endpoint data
HIV	ART-free remission/post-treatment viral control without rebound [224]	Not yet reliably achieved; immunogenicity often observed
HPV-associated lesions	HPV clearance, CIN/HSIL regression, lesion resolution [129]	Several candidates show lesion regression/viral clearance, especially in early disease
HPV-associated cancers	ORR, PFS, OS, durable tumor response	Signals mainly in combination with checkpoint inhibitors
EBV-associated malignancies	EBV DNA reduction, tumor response, delayed progression, PFS/OS	Immune responses observed; clinical benefit remains limited/context-dependent

## Data Availability

Not applicable.
